# Occupational stress among farm and ranch operators in the midwestern United States

**DOI:** 10.1186/s12889-021-12053-4

**Published:** 2021-11-12

**Authors:** Sabrine Chengane, Cheryl L. Beseler, Ellen G. Duysen, Risto H. Rautiainen

**Affiliations:** Department of Environmental, Agricultural and Occupational Health, College of Public Health, 984388 Nebraska Medical Center, University of Nebraska Medical Center, Omaha, NE 68198-4388 USA

**Keywords:** Occupational stress, Classification tree, Musculoskeletal disorders, Sleep deprivation, Fatigue, Agriculture, Surveillance

## Abstract

**Background:**

This study used surveillance data from 2018 and 2020 to test the stability of work-related strain symptoms (high stress, sleep deprivation, exhaustion) with demographic factors, work characteristics, and musculoskeletal symptoms among farm and ranch operators in seven midwestern states of the United States.

**Methods:**

Cross-sectional surveys were conducted among farm and ranch operators in 2018 (*n* = 4423) and 2020 (*n* = 3492). Operators were asked whether, in the past 12 months, they experienced extended work periods that resulted in high stress levels, sleep deprivation, exhaustion/fatigue, or other work-related strain symptoms. Covariates included personal and demographic factors, work characteristics, number of injuries, work-related health conditions, and exposures on the operation. Summary statistics were tabulated for explanatory and outcome variables. The classification (decision) tree approach was used to assess what variables would best separate operators with and without reported strain symptoms, based on a set of explanatory variables. Regularized regression was used to generate effect estimates between the work strain variables and explanatory variables.

**Results:**

High stress level, sleep deprivation, and exhaustion were reported more frequently in 2018 than 2020. The classification tree reproduced the 2018 model using 2020 data with approximately 80% accuracy. The mean number of reported MSD symptoms increased slightly from 1.23 in 2018 to 1.41 in 2020. Older age, more time spent in farm work, higher gross farm income (GFI), and MSD symptoms in six body regions (ankles/feet, knees, lower back, neck, shoulders, wrists/hands) were associated with all three work strain symptoms.

**Conclusions:**

Musculoskeletal pain and discomfort was a strong predictor for stress, sleep deprivation, and exhaustion among farmers and ranchers. This finding indicates that reducing MSD pain and discomfort is beneficial for both physical and mental health.

**Supplementary Information:**

The online version contains supplementary material available at 10.1186/s12889-021-12053-4.

## Background

Stress is the response to a perceived conflict between work demands and a person’s expectations, resources, and capacities [1]. An event that is internalized as a stressor in one person may not be a stressor in another and may depend on the presence of additional stressors [2]. Stress reactivity, the subjective emotional and physiological reaction to stress, is what we attempt to measure [3, 4]. Currently, there are no assessment scales that specifically target occupational stress in farm and ranch operators. The most frequently used scale with well-established psychometric properties is the Perceived Stress Scale [5], which covers psychosocial stress. If a valid and reliable occupational stress scale were designed, it would most likely not transfer to agricultural operators due to their disparate work environment.

The Stress Process Model describes how sources of stress produce manifestations of stress through mediators in the pathway [6]. Sources of stress include both eventful experiences and chronic life strains that occur with changes in self-concept. The mediators of stress include coping and social support. The model captures important aspects of the stress process in farmers. Although Pearlin et al. uses loss of employment as a source of stress in their 1981 study, disruptions to a farmer’s work including injury and compromised health are major contributors to stress in farmers [7, 8]. In the background are the economic challenges in farming that have existed for decades, creating chronic strain [9–11]. Changes in self-concept such as loss of self-esteem and feelings of failure are a source of stress that may lead to depression and are likely to partly explain the high suicide rate in farmers [12–16]. Continual readjustment to sources of stress such as work disruptions can cause chronic exhaustion [6]. Exhaustion is an “extreme tiredness” resulting from mental and physical fatigue, work hours, and an unfavorable environment [9, 17]. Exhaustion is one of the three dimensions of burnout that develops over time from living under chronic stress [9, 18]. Disturbed sleep is a consequence of stress and manifests as changes in sleep duration, initiation and maintenance [19, 20].

A farmer’s work differs from other occupational groups because, although they have autonomy over their work activities, they have no control over their larger work environment. Success is not only a function of a strong work ethic, but also of market prices, trade wars, subsidies, and the weather. There may also be additional challenges related to living in rural areas such as isolation and lack of healthcare services [21, 22]. Life strain, including economic and environmental uncertainty, creates instability leading to greater stress, disordered sleep, mood disorders and metabolic changes [19, 20, 23]. Left unabated, the burden of strain can lead to deviations from physiological homeostasis or “allostatic overload”, resulting in hormonal changes. Stress results in chronically elevated glucocorticoid levels, reduced glucose uptake, increased insulin production and promotion of body fat storage [24–26]. Stress, sleep disorders, obesity and depression are etiological agents that increase the risk of diabetes and other chronic diseases, creating additional work disruptions and exacerbation of the original stressors.

For the reasons stated above, farmers do not fit the organizational paradigm of job demand, job control, and social support at work as determinants of job stress [22, 27–30]. However, farmers are similar to other occupational groups in terms of the physiological responses to stress. In an occupational study of 174 middle-managers in a manufacturing company, perceived stress and mental and physical health outcomes, depression, anxiety, musculoskeletal, and cardiovascular complaints were more frequent in those with greater perceived stress, but no associations with sleep problems or exhaustion were reported [31]. In a study of 130 nurses, heightened job stress after installing technology in the workplace resulted in increased complaints of back pain and unfavorable work conditions [32]. Studies examining the relationship of stress specifically related to farm work, and correlates of stress such as sleep disorders and exhaustion have not been addressed simultaneously in a large group of farm/ranch operators. Work strain has often been limited to physical exertion and its physiological consequences [33, 34], however, mental work strain associated with stress, sleep and exhaustion are important factors when considering work strain [23, 35].

Using two years of cross-sectional surveillance data, this study aimed to describe demographic and work-related factors associated with self-reported high levels of stress, sleep deprivation, and exhaustion among farm and ranch operators in seven states in the Midwest. The brief survey conducted in 2018 and 2020 was part of an injury surveillance system. Therefore, a full investigation into stress, sleep and exhaustion was not undertaken in the short survey. Our interest was in identifying important correlates of mental work strain indicators and determining the robustness of the indicators.

Our specific research questions were (1) what are the most important factors associated with high levels of stress, sleep deprivation and exhaustion in a Midwest farming sample; (2) how do the important correlates of mental work strain symptoms differ in each of the three outcomes or can they be collapsed into an overall measure of mental work strain; and (3) are the most important indicators of these strain variables sufficiently robust that they can be replicated in a second, independent sample of farmers. We used a classification tree methodology to answer these three questions and hypothesized that the three measures were interchangeable with the same or similar risk factors. We further hypothesized that factors used to classify these strain outcomes would reproduce in an independent sample. We expected to see that the number of injuries would be a defining feature of the classifications. The findings of this study were expected to provide insight into how stress, sleep and exhaustion are related in farmers and ranchers and provide a basis for further large-scale studies on measures of chronic work strain in this occupational group.

## Methods

### Sample

In the spring and summer of 2018, a cross-sectional survey was conducted by the Central States Center for Agricultural Safety and Health (CS-CASH) in farm and ranch operators in a seven-state region (Iowa, Kansas, Minnesota, Missouri, Nebraska, North Dakota, and South Dakota). The survey was distributed to a random sample of 2500 farm and ranch operations in each of the seven states. The 29-question, paper-based Farm and Ranch Health and Safety Survey (FRHSS), created by CS-CASH, focused on injuries, chronic health outcomes, work-related exposures, and the use of personal protective equipment for up to three main operators working on a farm or ranch (Supplementary File 1). The survey was divided into seven sections on operators’ demographics, acute injuries, chronic health conditions, family members, hired workers, exposures, and a comment section. One question was related to mental work strain symptoms under the section addressing chronic health conditions of each operator. All the responses were received by mail and entered into REDCap. The study was determined to be exempt from human subjects’ research by the University of Nebraska Medical Center’s Institutional Review Board. We informed participants about the study with an accompanying letter. No signed waiver of consent was required by the University of Nebraska Medical Center Institutional Review Board (No. 452–11-EX).

Farm Market iD (FMiD) was the source of the sampling frame. A stratified random sample of 2500 farms in each of the seven states was selected for sampling. FMiD is a for-profit organization that creates farm databases from the United States Department of Agriculture (USDA) annual surveys and other public and private sources. The FMiD database covers 95% of agricultural producers in the United States. Farm and ranch operations with an email address and an estimated gross farm income of at least $5000 were included in the random sample. The random sample of 16,826 farm and ranch operations were contacted by letter in May of 2018, asking them to complete and return the survey form. A follow-up mailing to nonresponders was sent out in July, 2018, and data collection continued until November, 2018. These efforts resulted in a response rate of 19% including 3268 farms and ranches and 4423 individual operators. Additional detail on the 2018 data collection process can be found in Du et al., (2021) [36] A previous analysis of nonresponse bias in the 2018 sample found no evidence that responders differed from nonresponders on farm or ranch characteristics [37]. Responders were more likely to be married than nonresponders, but no other demographic differences were identified. The unit of analysis in this study is the individual operator.

Using the same sampling frame and methods, the initial mailing for the 2020 survey was sent out in March and the follow-up request was mailed out in June. The 2736 farms that responded represented 3492 individual farmers and ranchers. The response rate in 2020 was 16%. The 2020 surveys were deployed during the COVID-19 pandemic. COVID-19 appears to have elevated mental distress and worsened the preexisting farmers’ stress caused by issues of food supply chain, living in a rural area, and natural disasters [38–40].

### Measures

The primary outcome of interest was the response to the stem question: “Did the operator experience extended work periods that resulted in any of the following during the past 12 months?”, and respondents were asked to check all boxes that applied to them. The possible responses to this question were: none, high stress level, sleep deprivation, exhaustion/fatigue, and other, specify. The outcome variables were coded as 0 = not checked and 1 = checked for each of the three symptoms. Since no definitions were provided to participants, the responses reflect self-perception of their levels of stress, sleep deprivation, and exhaustion.

The personal and demographic covariates included age measured as a continuous variable, gender (male or female), gross farm income (GFI), primary occupation (50% of time doing farm/ranch work or other work), and the percent of time the operator spent working on the farm or ranch in the past 12 months in five categories (0–24%, 25–49%, 50–74%, 75–99, 100%). Work characteristics included whether the operation was a farm, ranch, or both coded in three categories; the number of injuries reported by the operator in the past 12 months (0, 1, 2, 3 or more); whether the operator sought medical care for an injury (yes/no); presence of musculoskeletal disorder (MSD) (yes/no), skin diseases (yes/no) or respiratory diseases (yes/no); and exposures on the operation (noise, skin, respiratory, and musculoskeletal) with each coded as yes or no.

We assessed MSD to specific body parts with the stem question “Did the operator experience pain or discomfort that affected his/her work in any of the following body areas during the past 12 months?” and again farmers/ranchers were asked to mark all that applied to them. We asked specifically about discomfort in the ankles/feet, elbow, hip/thigh, knees, low back, neck, shoulder area, and wrist/hands. These were coded as 0 = not present and 1 = present.

### Statistical analysis

We calculated summary statistics for the independent explanatory variables and for the three outcomes in the 2018 training and 2020 testing samples for the purpose of understanding potential differences in the test and training samples. The chi-square statistic was used to test for significant differences in the two independent samples. Tetrachoric correlations were used to calculate correlations between the binary strain variables in years 2018 and 2020. We calculated the proportion of variance explained when combining these three outcomes into a single measure of mental work strain. Polychoric correlations were used to calculate correlations between the ordinal injury variable (four levels) and each strain variable for the years 2018 and 2020.

Due to our three outcomes of interest being highly correlated binary variables for high stress, sleep deprivation and exhaustion, we chose to use a classification (decision) tree approach to assess what variables could best separate those with a reported strain from those without based on a set of explanatory variables. The classification tree partitions recursively into rectangles based on a specified loss function with the goal of minimizing the loss function with each sequential partition [41–43]. It is a simple algorithm that divides up the dimensional space into the most homogeneous groups of points using a greedy algorithm. Once a partition is made by splitting on a covariate, it is retained as additional splits are made on additional variables.

We used the classification model separately with each mental strain variable as the outcome. We trained the model with the 2018 FRHSS sample and tested the model in the 2020 FRHSS sample. We chose the Gini Index for the loss function because it is commonly used and produces reliably pure nodes [41]. The Gini Index is a loss function that calculates the probability of obtaining a purer separation of points every time the sample space is split into two regions by a variable value. The goal is to minimize the errors made by misclassifying a point into a specific partition. We used bootstrap aggregation to improve empirical estimation of uncertainty without assuming asymptotic normality and bootstrapped 500 trees for each outcome. We generated confusion matrices and calculated the misclassification rates for each final model. The importance of the variables used to classify those with and without a strain indicator was based on which of the variables contributed the most to improving the classification in the test data using the training model. The horizontal axis of the importance graph is the average decrease in the Gini Index averaged over 500 trees after splitting on a specified variable. Dendrograms are not produced using this approach because the algorithm averages over all 500 simulated trees in order to reduce the variance without increasing bias in the estimates.

These methods require complete cases for all observations so 130 observations were excluded from the training data due to missing responses on gender, number of injuries, percent time spent on farm work and primary occupation. For consistency, we also kept only complete observations in the test data where the same variables had missing values with the addition of GFI, which was missing on 14 operations. This reduced the test dataset by 204 observations. Our final training data set contained 4293 operators and the final testing data set contained 3288 operators.

Lastly, we examined MSD-affected body regions. Although not specifically related to our three primary aims, MSD was an important factor in work strain identified in the classification tree. The result needed further exploration. In our survey, operators were asked to check boxes for any MSD that they experienced in different areas of their bodies; operators could report up to eight MSD-affected regions. We first used lasso regularized regression with a binomial link function to generate effect estimates between sleep deprivation, high stress, and exhaustion on the individual body parts affected. The continuous variables age and GFI were standardized. In contrast to classical regression methods, regularized regression handles collinearity because of the algorithm used to estimate the effects [44, 45]. Similar to other machine learning algorithms, the most important variables can be identified, even with a lack of independence between the predictors. After variable selection, the models were refitted using separate logistic regression models for each of the three strain indicators and for each year of data for a total of six models. The lasso regularized regression analyses were done in the R package glmnet.

## Results

Differences in personal and work-related characteristics in the 2018 (*n* = 4293) and 2020 (*n* = 3288) samples are shown in Table 1. Farmers were younger in the 2018 sample compared to the 2020 sample (56.4 vs. 59.6, *p* < 0.0001). GFI was significantly lower in the 2020 sample (median = 121,812) compared to the 2018 sample (median = 392,434). Statistically significant differences were observed by type of farm, gender, primary occupation, percent of time spent working on the farm, number of injuries reported, presence of a respiratory disorder, and all exposures assessed (musculoskeletal, respiratory, skin and noise). Given the large sample size, the *p*-value is likely to be significant even when differences may not be meaningful. No statistically significant changes were observed for reported musculoskeletal discomfort or diagnosed skin conditions.

**Table 1:** Demographic and work-related characteristics in Farm and Ranch Health and Safety Survey respondents by year

Demographic and Work Characteristics	2018 Sample (*N* = 4293) n (%)	2020 Sample (*N* = 3288) n (%)
Type of Agricultural Production Operation ****		
Farm	3582 (83.4)	2153 (65.5)
Ranch	474 (11.0)	372 (11.3)
Both	237 (5.5)	763 (23.2)
Gender **		
Male	3691 (86.0)	2742 (83.4)
Female	602 (14.0)	546 (16.6)
Primary Occupation ****		
50% or more time spent farming/ranching	3600 (83.9)	2289 (69.6)
50% or more time spent on other work	693 (16.1)	999 (30.4)
Percentage of time spent working on the farm or ranch ****		
0–24%	248 (5.8)	455 (13.8)
25–49%	455 (10.6)	573 (17.4)
50–74%	445 (10.4)	429 (13.1)
75–99%	855 (19.9)	549 (16.7)
100%	2290 (53.3)	1282 (39.0)
Number of farm-related injuries in the past 12 months *		
None	3751 (87.4)	2905 (88.3)
One	449 (10.5)	304 (9.3)
Two	72 (1.7)	49 (1.5)
Three or more	21 (0.5)	30 (0.9)
Was doctor visit required for most serious injury?		
Yes	347 (8.1)	251 (7.6)
No	3946 (91.9)	3037 (92.4)
Pain or discomfort that affected work in past 12 months		
Yes	2736 (63.7)	2150 (65.4)
No	1557 (36.3)	1138 (34.6)
Musculoskeletal exposures in past 12 months ***		
Yes	3445 (80.2)	2532 (77.0)
No	848 (19.8)	756 (23.0)
Diagnosed respiratory disorder *		
Yes	991 (23.1)	833 (25.3)
No	3302 (76.9)	2455 (74.7)
Respiratory exposures in past 12 months ****		
Yes	2857 (66.6)	1.910 (58.1)
No	1436 (33.4)	1378 (41.9)
Diagnosed skin condition in past 12 months ***		
Yes	829 (19.3)	681 (20.7)
No	3464 (80.7)	2607 (79.3)
Exposed to chemicals or animal-based allergens in past 12 months		
Yes	3441 (80.1)	2516 (76.5)
No	852 (19.9)	772 (23.5)
Noise exposure ****		
Yes	3671 (85.5)	2644 (80.4)
No	622 (14.5)	644 (19.6)
High Stress ****		
Yes	1235 (28.8)	713 (21.7)
No	3058 (71.2)	2575 (78.3)
Sleep Deprivation ****		
Yes	1046 (24.4)	604 (18.4)
No	3247 (75.6)	2684 (81.6)
Exhaustion ****		
Yes	1259 (29.3)	754 (22.9)
No	3034 (70.7)	2534 (77.1)

****p < 0.0001, ***p < 0.001, **p < 0.01, *p < 0.05

Operators endorsing any of the strain indicators were younger than those who did not in 2018 and 2020, but the mean difference was only five years. GFI was only slightly higher in those who endorsed any of the strain indicators in 2018 but showed significant differences in 2020. Those with higher GFI were more likely to report high stress, sleep deprivation and exhaustion.

Reported levels of high stress, sleep deprivation and exhaustion were also significantly different from 2018 to 2020 (*p* < 0.0001). Interestingly, reported strain indicators were significantly lower in 2020 than in 2018, despite the coronavirus pandemic that impacted nearly all during 2020. In the 2018 sample the correlation between stress and sleep was 0.74, between stress and exhaustion 0.68, and between exhaustion and sleep 0.69. These three indicators combined explained 71% of the variance in work strain. The correlations remained similar in the 2020 sample (stress and sleep = 0.70, stress and exhaustion = 0.66, exhaustion and sleep = 0.71; 69% of the variance explained). The correlation between number of injuries and the strain variables increased from 2018 to 2020 (sleep 0.21 to 0.26, stress 0.25 to 0.32, exhaustion 0.26 to 0.36).

**Fig. 1:**
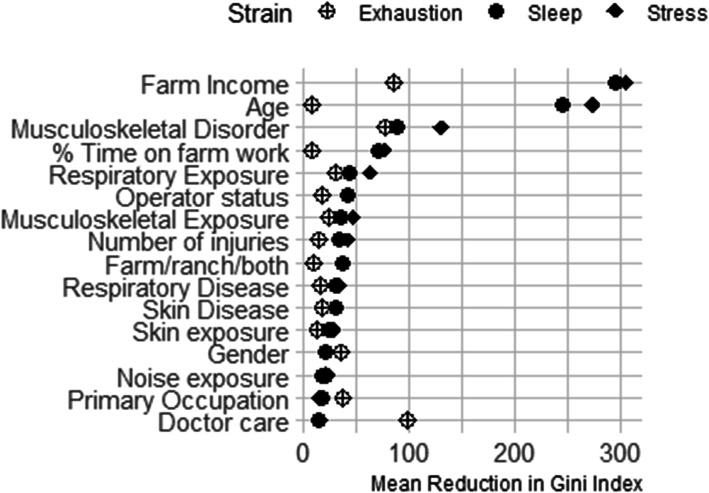
Importance plot of demographic and farm characteristics used to classify stress, sleep deprivation or exhaustion. The classification tree was trained on a sample of 4293 from the 2018 Farm and Ranch Health and Safety Survey and tested in 3288 in the 2020 survey sample

The classification tree analysis revealed that high stress and sleep deprivation were strongly related to GFI, but exhaustion was not (Fig. 1). Age was the second most important variable for sleep deprivation and high stress, but not for exhaustion. Almost equally, exhaustion was related to seeking a doctor’s care for an injury and GFI. Experiencing an MSD was an important classifier for all three indicators. The misclassification rate for each of the three indicators was nearly the same. Sleep was the lowest at 20%, and stress and exhaustion were equal at 22%. We classified farmers on high stress, sleep deprivation and exhaustion with a success rate of approximately 80% using these 16 demographic and work-related variables.

Due to the observation that MSD was important to correctly classifying the strain categories beyond age and GFI, we wanted to explore further what might be driving this finding. We examined work-related sleep deprivation and high stress because of the theoretical causal pathway due to MSD increasing work stress and reducing sleep quality. In addition, we examined exhaustion, but viewed it as an outcome of stress and sleep deprivation in the Stress Process Model [6]. In 2018, the mean number of MSD-affected regions reported was 1.23 (SD = 1.50). In 2020, the mean number of MSD-affected regions increased to 1.41 (SD = 1.64) (*p* < 0.0001), however, 95% of the participants reported four or fewer body regions with pain or discomfort in 2018 and 2020. The Cohen’s D for effect size was only 0.11, a small effect, and is not likely to be clinically significant.

The regression results showed that MSD was similar in those with sleep deprivation and high stress, but only in 2018 (Table 2). The discomfort was substantial for all body regions except the elbows and hip/thigh areas in the 2018 sample for both stress and sleep outcomes. Statistically significant odds ratios ranged from 1.18 to 1.98 and were highest for wrist/hand discomfort. Percent time spent working on the farm/ranch, age, and GFI were also important explanatory effects. The 2020 sample showed fewer associations between MSD and strain indicators. High stress was significantly associated with low back, neck, shoulder, and wrist/hand discomfort, as well as age and time spent in farm work. Sleep deprivation was significantly associated only with shoulder discomfort, time spent doing farm work, age, and GFI. Time spent working on the farm, GFI, and age were consistently negatively associated with the strain indicators (Table 2). Exhaustion was significantly associated with nearly all of the MSD regions in 2018 and 2020 with similar effect sizes (ORs 1.27–2.00). Only feet and ankle problems was not associated with exhaustion in 2020 (Table 2). Operators who reported high stress, sleep deprivation and/or exhaustion spent less time doing farm work, worked on farms and/or ranches with a lower GFI and were younger in age than those who did not report these work strains. The differences in these MSD variables by year might account for some of the misclassification error in the classification tree models.

**Table 2:** Refitted logistic regressions for stress, sleep deprivation, and exhaustion in 2018 (n = 4293) and 2020 (n = 3288)

Musculoskeletal discomfort by body region	2018		
	High stress level	Sleep deprivation	Exhaustion
	Odds Ratio (95% CI)	Odds Ratio (95% CI)	Odds Ratio (95% CI)
Ankles/feet	1.73 (1.40, 2.12)	1.72 (1.40, 2.12)	2.00 (1.63, 2.45)
Knees	1.33 (1.12, 1.56)	1.20 (1.02, 1.42)	1.43 (1.22, 1.68)
Lower back	1.43 (1.22, 1.67)	1.32 (1.13, 1.55)	1.44 (1.23, 1.67)
Neck (cervical spine)	1.84 (1.48, 2.28)	1.67 (1.34, 2.07)	1.68 (1.36, 2.08)
Shoulders	1.49 (1.26, 1.76)	1.18 (0.99, 1.39)	1.49 (1.26, 1.75)
Wrists/hands	1.76 (1.42, 2.17)	1.98 (1.60, 2.45)	1.59 (1.28, 1.96)
Percent time spent working on farm	0.52 (0.49, 0.54)	0.49 (0.47, 0.52)	0.53 (0.51, 0.56)
Age	0.67 (0.62, 0.72)	0.68 (0.63, 0.73)	0.81 (0.75, 0.87)
Gross farm income	0.91 (0.84, 0.97)	0.87 (0.81, 0.94)	0.83 (0.77, 0.90)
Musculoskeletal discomfort by body region	2020		
	High stress level	Sleep deprivation	Exhaustion
	Odds Ratio (95% CI)	Odds Ratio (95% CI)	Odds Ratio (95% CI)
Ankles/feet	Not Selected	Not Selected	Not Selected
Knees	Not Selected	Not Selected	1.31 (1.08, 1.60)
Lower back	1.40 (1.18, 1.67)	Not Selected	1.39 (1.16, 1.65)
Neck (cervical spine)	1.30 (1.03, 1.63)	Not selected	1.27 (1.01, 1.59)
Shoulders	1.51 (1.23, 1.85)	2.16 (1.80, 2.60)	1.38 (1.12, 1.68)
Wrists/hands	1.63 (1.27, 2.09)	Not Selected	1.69 (1.33, 2.16)
Percent time spent working on farm	0.51 (0.49, 0.54)	0.51 (0.49, 0.54)	0.54 (0.51, 0.57)
Age	0.65 (0.59, 0.70)	0.70 (0.64, 0.77)	0.76 (0.70, 0.83)
Gross farm income	Not Selected	0.83 (0.77, 0.91)	Not Selected

## Discussion

The 16 demographic and work characteristics used in our models classified individuals correctly on the presence of high stress, sleep deprivation and exhaustion about 80% of the time. Despite differences in the two samples on important characteristics, the 2018 model was largely replicated with the 2020 data. Although there is overlap in all three mental strain indicators, as shown by their high correlations, there are distinct differences in the factors that discriminate between them. The stress and sleep outcomes were more similar to one another in their defining characteristics than they were to exhaustion. This is what might be expected under the Stress Process Model since exhaustion would be the result of chronic stressors [6]. Finances, current pain and discomfort, and farm exposures that operators face every day are likely to be more closely linked to stress and sleep. Exhaustion would be a longer term outcome, an effect which would not be seen in cross-sectional data but might be a result of a serious work injury that causes long-term disruptions to work.

GFI and age were the strongest classifiers of those reporting high stress levels and sleep deprivation. Financial challenges have long been a primary stressor in the farming community [7, 8, 22, 46]. Previous studies have shown that younger farmers experience more stress [7, 45, 46] possibly because they do not have the capital of older farmers, have greater debt loads, and may be working on jobs off the farm. Age is likely to be a surrogate for available resources, which can mitigate stress levels.

After GFI and age, MSD was the next most important discriminator of those reporting high stress, sleep deprivation and exhaustion, but was strongest for stress and exhaustion. MSD is associated with physical work strain due to improper lifting, carrying heavy loads, poor posture, repetitive motions, exposure to vibration and other physical activities that strain and contort the musculoskeletal system. At 31%, low back pain was the most frequently reported work-related MSD in Irish farmers [47]. However, MSD might be a manifestation of mental stress in addition to physical stress. Back pain was a strong indicator of stress in farmers in a 1987 study of 407 male farm operators [48]. Back pain and other MSD issues have been reported in organizational studies of perceived stress, even when physical work strain is minimal, such as in managers, or adjusted for in models [23, 31, 35]. A 2007 survey of 6091 workers in four Swiss companies showed that general stress was associated with back, neck, and shoulder pain, after adjusting for physical work strain, time spent working and time pressures at work [49]. Our modeling of the individual regions affected by MSD showed that six regions were strongly and consistently associated with stress, sleep and exhaustion in 2018. The strongest associations were observed for wrists and hands, which are critical to the work farmers do. Pain in the hands or wrists is likely to increase stress because it is a disruption to work.

The associations with MSD dramatically differed in the 2020 sample for sleep deprivation and high stress. Indicators of stress including back, neck and shoulder pain were elevated, but back pain was not important in separating the sleep deprived from those who were not. Wrist and hand discomfort was strongly associated with stress but was not selected by the regularized regression in the sleep model. There was a slightly higher number of MSD regions reported in 2020 compared to 2018, which makes the narrowing of the body regions affected by MSD perplexing.

Time spent working on the farm contributed to partitioning operators into the high stress and sleep deprived categories. Possible reason for this is the decades-long observation that farmers who need to work off the farm do not see themselves as successful compared to those who do not need to work off the farm, especially when the off-farm job is part-time [50]. Working off the farm can worsen the problems of time pressures and work overload, thereby increasing stress [9, 22]. This is more likely to be true for younger farmers/ranchers than older ones. One of the primary concerns of farmers is the time pressures during their busy seasons. Work time was not as strong a classifier in the exhaustion model compared to the stress and sleep models, which seems counter-intuitive since work overload should increase exhaustion. However, with farmers, being able to work more and stay healthy might decrease stress levels and improve needed sleep during the busy seasons.

Respiratory exposures and exposures to physical work that can cause MSD were also discriminatory between those with and without a mental work strain. This result confirms the stress studies in farmers where hazardous working conditions is stated as being a stressor [22, 51]. It was expected that farm-related injuries would be a strong classifier variable for high stress [52], but it was similar to operator status in importance. It might be that the type of injury is what increases stress and results in sleep deprivation. Injuries that disrupt work over extended periods of time are probably more likely to cause distress than injuries that heal quickly with a full recovery.

Many of the variables identified in the decision tree analysis have been previously included in farm stress studies for decades. In the 2018 FRHSS data, Yu et al., (2021) showed that high stress, sleep deprivation and exhaustion showed the strongest effects on MSD [36]. The current study replicates this finding, but also shows the robustness of the finding by showing it is reproducible in an independent sample of operators. The novel contribution this approach makes to the literature is the importance of MSD as a discriminatory feature of high stress, sleep deprivation and exhaustion. The tree method identified a subgroup of MSD-affected farmers/ranchers with mental work strain. It is not known whether some of this work strain is due to psychological strain, physical strain, or both. Previous studies suggest it may be of both types. Realizing how many work stressors operators face, the test data fit the training model relatively well, even though there were differences in the 2018 and 2020 sample characteristics for reasons that are not clear. Possible explanations are simply variation in sampling and responses, or an effect of the COVID-19 pandemic. Further investigation is warranted to assess what might be causing these differences across survey years that were only two years apart but were very different types of years. Stress and its related effects may have been worse for farmers due to being at the height of trade wars with China that began in early 2018 and lasted 18 months. This situation improved in 2020, and then the pandemic happened.

This study has a number of limitations. The three mental work strain variables were each measured with a single item on a brief questionnaire although these are complex, multidimensional constructs. It is probably reasonable to assume that farmers and ranchers know how stressed, sleep deprived and exhausted they feel when overworked, but these are self-reported and self-perceived without biological data or any type of personal journal to validate the observations. The decision tree also has limitations. Once the algorithm makes a split on a variable, the split is retained. The algorithm does not generate effect sizes or inform the directionality of the effects. These analyses must be pursued to understand the meaning of the partitioning. In our analyses, operations with higher GFI had operators with a greater frequency of strain indicators but in the MSD models the GFI effect was reversed, e.g., lower GFI was associated with greater stress and sleep deprivation. These observations need to be explored further.

## Conclusions

Musculoskeletal discomfort might be a stronger indicator of mental work strain than other work variables for stress and sleep disorders. Psychological strain could be adding to the physical strains that agricultural operators experience, especially in younger farmers, and adding to the MSD burden. Healthcare providers might consider addressing pain and discomfort in their agricultural communities as a measure of both physical and mental health and talk to their patients about their work stresses.

## Supplementary Information

**Additional file 1.** Farm and Ranch Health and Safety Survey. The Farm and Ranch Health and Safety Survey was created by the author, Rautiainen, for purposes of surveillance. Survey form mailed to a stratified (by state, 7 US states) random sample of farm and ranch operators with a postage paid return envelope, with one repeat to non-respondents. The survey form requested information for up to three operators (farmers and ranchers and family members participating in the operation of the agricultural enterprise) on injuries, chronic health conditions, work exposures, and protective practices. Additional variables were merged on the operation level regarding crop and livestock production from the Farm Market iD database (private company specializing in providing agricultural production data)

## Data Availability

Data are available from the corresponding author upon reasonable request.

## References

[1] Karasek RA, Theorell T (1990). Healthy work: stress, productivity, and the reconstruction of working life.

[2] Federenko IS, Schlotz W, Kirschbaum C, Bartels M, Hellhammer DH, Wüst S (2006). The heritability of perceived stress. Psychol Med.

[3] Kupper N, Jankovic M, Kop WJ (2021). Individual differences in cross-system physiological activity at rest and in response to acute social stress. Psychosom Med.

[4] Kelly S, Hertzman C, Daniels M (1997). Searching for the biological pathways between stress and health. Annu Rev Public Health.

[5] Cohen S, Kamarck T, Mermelstein R (1983). A global measure of perceived stress. J Health Soc Behav.

[6] Pearlin LI, Menaghan EG, Lieberman MA, Mullan JT (1981). The stress process. J Health Soc Behav.

[7] Yazd SD, Wheeler SA, Zuo A (2019). Key risk factors affecting farmer’s mental health: a systematic review. Int J Environ Res Public Health.

[8] Kearney GD, Rafferty AP, Hendricks LR, Allen DL, Tutor-Marcom R (2014). A cross-sectional study of stressors among farmers in eastern North Carolina. N C Med J.

[9] Truchot D, Andela M (2018). Burnout and hopelessness among farmers: the farmers stressors inventory. Soc Psychiatry Psychiatr Epidemiol.

[10] Thompson EA, McCubbin HI (1987). Farm families in crisis: an overview of resources. Fam Relat.

[11] Rosenblatt PC, Keller LO (1983). Economic vulnerability and economic stress in farm couples. Fam Relat.

[12] Thomas HV, Lewis G, Thomas DR, Salmon RL, Chalmers RM, Coleman TJ, Kench SM, Morgan-Capner P, Meadows D, Sillis M, Softley P (2003). Mental health of British farmers. Occup Environ Med.

[13] Peterson C, Stone DM, Marsh SM, Schumacher PK, Tiesman HM, LiKamWa McIntosh W, Luo F (2018). Suicide rates by major occupational group - 17 states, 2012 and 2015. MMWR..

[14] Kennedy A, Cerel J, Kheibari A, Leske S, Watts J (2021). A comparison of farming- and non-farming-related suicides from the United States’ National Violent Deaths Reporting System, 2003–2016. Suicide Life Threat Behav.

[15] Fraser CE, Smith KB, Judd F, Humphreys JS, Fragar LJ, Henderson A (2005). Farming and mental health problems and mental illness. Int J Soc Psych.

[16] Perceval M, Kolves K, Reddy P, De Leo D (2017). Farmer suicides: a qualitative study from Australia. Occup Med.

[17] Botha N, White T. Distress and burnout among NZ dairy farmers: Research findings and policy recommendations. Paper presented at APEN international conference, Transformative Change: Chosen or Unchosen pathways to innovation, resilience and prosperity, 26–28 August 2013, Christchurch, New Zealand, Christchurch, New Zealand.

[18] Kallioniemi MK, Simola A, Kaseva J, Kymäläinen HR (2016). Stress and burnout among Finnish dairy farmers. J Agromedicine.

[19] Hirotsu C, Tufik S, Andersen ML (2015). Interactions between sleep, stress, and metabolism: from physiological to pathological conditions. Sleep Sci.

[20] Akerstedt T (2006). Psychosocial stress and impaired sleep. Scand J Work Environ Health.

[21] Brew B, Inder K, Allen J, Thomas M, Kelly B (2016). The health and wellbeing of Australian farmers: a longitudinal cohort study. BMC Public Health.

[22] McShane CJ, Quirk F, Swinbourne A (2016). Development and validation of a work stressor scale for Australian farming families. Aust J Rural Health.

[23] Leineweber C, Kecklund G, Orth-Gomer K (2007). Prediction of cardiocerebrovascular and other significant disease from disordered sleep and work strain. Scand J Work Environ Health.

[24] Guidi J, Lucente M, Sonino N, Fava GA (2021). Allostatic load and its impact on health: a systematic review. Psychother Psychosom.

[25] McEwen BS, Wingfield JC (2003). The concept of allostasis in biology and biomedicine. Horm Behav.

[26] McEwen BS (2000). 2000. Allostasis and allostatic load: implications for neuropsychopharmacology. Neuropsychopharmacology..

[27] Rusli BN, Edimansyah BA, Naing L (2008). Working conditions, self-perceived stress, anxiety, depression and quality of life: a structural equation model approach. BMC Public Health.

[28] Dollard MF, Winefield HR. A test of the demand-control/support model of work stress in correctional officers. J Occup Health Psychol. 1998;3(3):243:264. 9684215

[29] Bourbonnais R, Comeau M, Vezina M (1999). Job strain and evolution of mental health among nurses. J Occup Health Psychol.

[30] Karasek R (1979). Job demands, job decision latitude, and mental strain: implications for job redesign. Adm Sci Q.

[31] Limm H, Angerer P, Heinmueller M, Marten-Mittag B, Nater UM, Guendel H (2010). Self-perceived stress reactivity is an indicator of psychosocial impairment at the workplace. BMC Public Health.

[32] Jhun HJ, Cho SI, Park JT (2004). Changes in job stress, musculoskeletal symptoms, and complaints of unfavorable working conditions among nurses after the adoption of a computerized order communication system. Int Arch Occup Environ Health.

[33] Bostad E, Swensson C, Pinzke S. Physical working conditions in young cattle production in Sweden. 2013;19(1):19–J Agric Saf Health, 35. 10.13031/2013.42540. 10.13031/2013.42540 23600167

[34] Costa G, Berti F, Betta A (1989). Physiological cost of apple-farming activities. Appl Ergon.

[35] Augner C, Kaiser G (2019). Predictors of musculoskeletal symptoms in radiology technologists in Austria. Europe Work.

[36] Du Y, Baccaglini L, Johnson A, Puvvula J, Rautiainen RH. Factors associated with musculoskeletal discomfort in farmers and ranchers in the US central states. J Agromedicine, 1-13. 2021:1–13. 10.1080/1059924X.2021.1893880. 10.1080/1059924X.2021.1893880 33645460

[37] Beseler CL, Rautiainen R. Assessing nonresponse bias in farm injury surveillance data. J Agricultural Safety and Health. (in press). doi: 10.13031/jash.14554 10.13031/jash.14554 34729972

[38] Henning-Smith C, Tuttle M, Kozhimannil KB. Unequal distribution of COVID-19 risk among rural residents by race and ethnicity. J Rural Health 2021;37(1):224–226. 10.1111/jrh.12463. 10.1111/jrh.12463 PMC7273062 32396220

[39] Keeney AJ, Hernandez PJ, Meng Y. Assessing farm stress and community supports in a U.S.-Mexico border county. J Agric Saf Health. 2021;27(1):1–12. 10.13031/jash.14213. 10.13031/jash.14213 PMC8684411 34931114

[40] Usher K, Ranmuthugala G, Maple M, Dorkin J, Douglas L, Coffey Y, Bhullar N (2021). The 2019-2020 bushfires and COVID-19: the ongoing impact on the mental health of people living in rural and farming communities. Int J Ment Health Nurs.

[41] Krzywinksi K, Altman N (2017). Classification and regression trees. Nat Methods.

[42] Venkatasubramaniam A, Wolfson J, Mitchell N, Barnes T, JaKa M, French S (2017). Decision trees in epidemiological research. Emerg Themes Epidemiol.

[43] Breiman L (1984). Friedman JH.

[44] Zou H, Hastie T (2005). Regularization and variable selection via the elastic net. J Royal Stat Soc Ser B.

[45] Ogutu JO, Schulz-Streeck T, Piepho HP. Genomic selection using regularized linear regression models: ridge regression, lasso, elastic net and their extensions. BMC Proceedings. 2012;(6 Suppl 2):S10. 10.1186/1753-6561-6-S2-S10. 10.1186/1753-6561-6-S2-S10 PMC3363152 22640436

[46] Deary IJ, Willock J, McGregor M. Stress in farming. Stress Med. 1997;13(2):131–6. 10.1002/(SICI)1099-1700(199704)13:2<131::AID-SMI727>3.0.CO;2-T.

[47] Walker JL, Walker LJS. Self-reported stress symptoms in farmers. J Clin Psychol. 1988;44(1):10–6. 10.1002/1097-4679(198801)44:1<10::aid-jclp2270440103>3.0.co;2-6. 10.1002/1097-4679(198801)44:1<10::aid-jclp2270440103>3.0.co;2-6 3343357

[48] Walker LS (1987). Walker JL. Stresses and occupational symptoms predictive of distress in farmers Fam Relat.

[49] Hammig O, Knecht M, Laubli T, Bauer GF (2011). Work-life conflict and musculoskeletal disorders: a cross-sectional study of an unexplored association. BMC Musculoskelet Disord.

[50] Osborne A, Blake C, Meredith D, Kinsella A, Phelan J, McNamara J, Cunningham C (2013). Work-related musculoskeletal disorders among Irish farm operators. Am J Indust Med.

[51] Thu K, Lasley P, Whitten P, Lewis M, Donham KJ, Zwerling C, Scarth R (1997). Stress as a risk factor for agricultural injuries. J Agromedicine.

[52] Eberhardt BJ, Pooyan A (1990). Development of the farm stress survey: factorial structure, reliability, and validity. Educ Psychol Measurement.

