# Supplementary material for: Occupational stress among farm and ranch operators in the midwestern United States
Source: BMC Public Health. 2021 Nov 12;21:2076. doi: 10.1186/s12889-021-12053-4 (PMC8587493; doi:10.1186/s12889-021-12053-4)
Supplement: Supplementary file 1 — Additional file 1. Farm and Ranch Health and Safety Survey. The Farm and Ranch Health and Safety Survey was created by the author, Rautiainen, for purposes of surveillance. Survey form mailed to a stratified (by state, 7 US states) random sample of farm and ranch operators with a postage paid return envelope, with one repeat to non-respondents. The survey form requested information for up to three operators (farmers and ranchers and family members participating in the operation of the agricultural enterprise) on injuries, chronic health conditions, work exposures, and protective practices. Additional variables were merged on the operation level regarding crop and livestock production from the Farm Market iD database (private company specializing in providing agricultural production data) [file 12889_2021_12053_MOESM1_ESM.pdf]

Instructions
- Complete this form using a pen or pencil.
- Check entire box; print squarely in boxes and above lines.

Correct ☒ AB   Incorrect ☐ ☒ AB

SECTION 1 OPERATORS

1. Is your operation primarily a farm or a ranch? ☐ Farm ☐ Ranch ☐ Both

2. Please answer the following questions for up to three **primary operators** (individuals) on this operation as of today.

	Principal Operator	Operator 2	Operator 3
a. What is the operator's age?	☐☐ Years	☐☐ Years	☐☐ Years
b. Sex of operator?	☐ Male ☐ Female	☐ Male ☐ Female	☐ Male ☐ Female
c. At which occupation did the operator spend the majority (50% or more) of his/her worktime in the past 12 months?	☐ Farm/ranch work<br>☐ Other	☐ Farm/ranch work<br>☐ Other	☐ Farm/ranch work<br>☐ Other
d. What percentage of this operator's worktime was spent working on the farm/ranch in the past 12 months? (Mark only one.)	☐ 100%<br>☐ 75-99%<br>☐ 50-74%<br>☐ 25-49%<br>☐ 0-24%	☐ 100%<br>☐ 75-99%<br>☐ 50-74%<br>☐ 25-49%<br>☐ 0-24%	☐ 100%<br>☐ 75-99%<br>☐ 50-74%<br>☐ 25-49%<br>☐ 0-24%

SECTION 2 ACUTE INJURIES TO OPERATORS

Definitions: "Injury" is the result of a sudden, unexpected, forceful event, which has an external cause, and which results in bodily damage or loss of consciousness. "Farm-related" includes work and leisure activities on this operation, plus commuting, transport, and business trips for this operation.

	Principal Operator	Operator 2	Operator 3
3. How many farm-related injuries occurred to each operator during the past 12 months?	☐ None<br>☐ One<br>☐ Two<br>☐ Three or more	☐ None<br>☐ One<br>☐ Two<br>☐ Three or more	☐ None<br>☐ One<br>☐ Two<br>☐ Three or more

The following questions are about the **most serious injury** to each operator. (If no injuries in past 12 months, skip to Section 3.)

	Principal Operator	Operator 2	Operator 3
4. In what month did the most serious injury occur?	Month (1-12) ☐☐	Month (1-12) ☐☐	Month (1-12) ☐☐
5. Did the injury happen during farm-related work or leisure? (Mark only one.)	☐ Work<br>☐ Leisure	☐ Work<br>☐ Leisure	☐ Work<br>☐ Leisure
6. Where did this injury occur? (Mark only one.)	☐ Home/office<br>☐ Farm building<br>☐ Farm yard<br>☐ Field/pasture<br>☐ Road/off-farm	☐ Home/office<br>☐ Farm building<br>☐ Farm yard<br>☐ Field/pasture<br>☐ Road/off-farm	☐ Home/office<br>☐ Farm building<br>☐ Farm yard<br>☐ Field/pasture<br>☐ Road/off-farm

	Principal Operator	Operator 2	Operator 3
7. What was the primary object or substance that caused this injury? (Mark only one).	☐ Tractor<br>☐ ATV<br>☐ Machinery<br>☐ Truck/other vehicle<br>☐ Power tool<br>☐ Hand tool<br>☐ Building/structure<br>☐ Ground/floor/surface<br>☐ Livestock<br>☐ Other, specify:	☐ Tractor<br>☐ ATV<br>☐ Machinery<br>☐ Truck/other vehicle<br>☐ Power tool<br>☐ Hand tool<br>☐ Building/structure<br>☐ Ground/floor/surface<br>☐ Livestock<br>☐ Other, specify:	☐ Tractor<br>☐ ATV<br>☐ Machinery<br>☐ Truck/other vehicle<br>☐ Power tool<br>☐ Hand tool<br>☐ Building/structure<br>☐ Ground/floor/surface<br>☐ Livestock<br>☐ Other, specify:
8. What body part was injured? (Mark all that apply.)	☐ Eye/head/neck<br>☐ Chest/trunk<br>☐ Back<br>☐ Arm/shoulder<br>☐ Finger<br>☐ Hand/wrist<br>☐ Leg/knee/hip<br>☐ Ankle/foot/toe<br>☐ Other, specify:	☐ Eye/head/neck<br>☐ Chest/trunk<br>☐ Back<br>☐ Arm/shoulder<br>☐ Finger<br>☐ Hand/wrist<br>☐ Leg/knee/hip<br>☐ Ankle/foot/toe<br>☐ Other, specify:	☐ Eye/head/neck<br>☐ Chest/trunk<br>☐ Back<br>☐ Arm/shoulder<br>☐ Finger<br>☐ Hand/wrist<br>☐ Leg/knee/hip<br>☐ Ankle/foot/toe<br>☐ Other, specify:
9. What professional medical care did this injury require? (Mark all that apply.)	☐ None<br>☐ Doctor/clinic visit<br>☐ Hospitalization	☐ None<br>☐ Doctor/clinic visit<br>☐ Hospitalization	☐ None<br>☐ Doctor/clinic visit<br>☐ Hospitalization
10. How much lost farm/ranch work time resulted from this injury? (Mark only one.)	☐ No lost time<br>☐ Less than 1/2 day<br>☐ 1/2 to 1 day<br>☐ 2 to 6 days<br>☐ 7-29 days<br>☐ 30 days or more	☐ No lost time<br>☐ Less than 1/2 day<br>☐ 1/2 to 1 day<br>☐ 2 to 6 days<br>☐ 7-29 days<br>☐ 30 days or more	☐ No lost time<br>☐ Less than 1/2 day<br>☐ 1/2 to 1 day<br>☐ 2 to 6 days<br>☐ 7-29 days<br>☐ 30 days or more
11. What were the estimated costs from this injury, including out-of-pocket costs and costs paid by insurance? (Mark '0' if none.)	Out-of-pocket $<br>Insurance paid $	Out-of-pocket $<br>Insurance paid $	Out-of-pocket $<br>Insurance paid $
12. Please describe how the injury happened. **Use space at the end of the survey for additional detail if needed.			

SECTION 3 CHILDREN, YOUTH AND FAMILY MEMBERS

13. How many children, youth and adult family members lived on this farm/ranch during the past 12 months? (Including operators listed in previous sections)

	Number of persons (mark '0' if none)	How many of them were injured? (mark '0' if none)
a. Children 0 - 9 years of age ------------------------------->		
b. Youth 10 - 19 years of age ------------------------------->		
c. Adults 20 - 64 years of age ------------------------------->		
d. Adults 65 years of age and older ------------------------->		

SECTION 4 HIRED WORKERS

14. How many hired farm workers, including paid family members and office workers -

	Number of persons (mark '0' if none)	How many of them were injured? (mark '0' if none)
a. Worked less than 150 days on this operation during the past 12 months?		
b. Worked 150 days or more on this operation during the past 12 months?		

SECTION 5 CHRONIC HEALTH CONDITIONS TO OPERATORS

	Principal Operator	Operator 2	Operator 3
15. **Respiratory diseases:** Has the operator ever been diagnosed by a physician with any of the following respiratory conditions? (Mark all that apply.)	☐ None<br>☐ Chronic obstructive pulmonary dis. (COPD)<br>☐ Asthma<br>☐ Farmers lung<br>☐ Sinus disease (Sinusitis)<br>☐ Nasal inflammation, runny nose (Rhinitis)<br>☐ Environ. allergies	☐ None<br>☐ Chronic obstructive pulmonary dis. (COPD)<br>☐ Asthma<br>☐ Farmers lung<br>☐ Sinus disease (Sinusitis)<br>☐ Nasal inflammation, runny nose (Rhinitis)<br>☐ Environ. allergies	☐ None<br>☐ Chronic obstructive pulmonary dis. (COPD)<br>☐ Asthma<br>☐ Farmers lung<br>☐ Sinus disease (Sinusitis)<br>☐ Nasal inflammation, runny nose (Rhinitis)<br>☐ Environ. allergies
16. **Hearing loss:** Does the operator have hearing loss? (diagnosed or self-assessed) (Mark only one.)	☐ None<br>☐ Mild<br>☐ Moderate<br>☐ Severe	☐ None<br>☐ Mild<br>☐ Moderate<br>☐ Severe	☐ None<br>☐ Mild<br>☐ Moderate<br>☐ Severe
17. **Skin diseases:** Has the operator ever been diagnosed by a physician with any of the following skin conditions? (Mark all that apply.)	☐ None<br>☐ Irritant dermatitis<br>☐ Allergic dermatitis<br>☐ Skin cancer<br>☐ Other, specify:	☐ None<br>☐ Irritant dermatitis<br>☐ Allergic dermatitis<br>☐ Skin cancer<br>☐ Other, specify:	☐ None<br>☐ Irritant dermatitis<br>☐ Allergic dermatitis<br>☐ Skin cancer<br>☐ Other, specify:
18. **Work strain symptoms:** Did the operator experience extended work periods that resulted in any of the following during the past 12 months? (Mark all that apply.)	☐ None<br>☐ High stress level<br>☐ Sleep deprivation<br>☐ Exhaustion/fatigue<br>☐ Other, specify:	☐ None<br>☐ High stress level<br>☐ Sleep deprivation<br>☐ Exhaustion/fatigue<br>☐ Other, specify:	☐ None<br>☐ High stress level<br>☐ Sleep deprivation<br>☐ Exhaustion/fatigue<br>☐ Other, specify:

	Principal Operator	Operator 2	Operator 3
19. **Musculoskeletal discomfort:** Did the operator experience pain or discomfort that affected his/her work in any of the following body areas during the past 12 months? (Mark all that apply.)	☐ None<br>☐ Neck<br>☐ Shoulder<br>☐ Upper back<br>☐ Elbows<br>☐ Wrists/hands<br>☐ Low back<br>☐ Hips/thighs<br>☐ Knees<br>☐ Ankle/feet	☐ None<br>☐ Neck<br>☐ Shoulder<br>☐ Upper back<br>☐ Elbows<br>☐ Wrists/hands<br>☐ Low back<br>☐ Hips/thighs<br>☐ Knees<br>☐ Ankle/feet	☐ None<br>☐ Neck<br>☐ Shoulder<br>☐ Upper back<br>☐ Elbows<br>☐ Wrists/hands<br>☐ Low back<br>☐ Hips/thighs<br>☐ Knees<br>☐ Ankle/feet

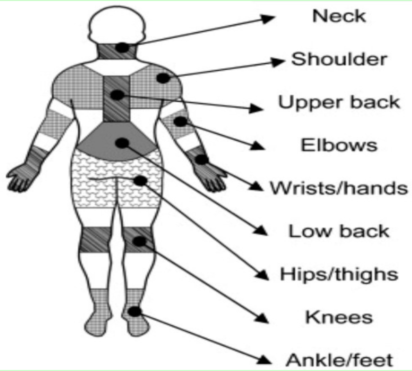

SECTION 6 EXPOSURES TO OPERATORS

	Principal Operator	Operator 2	Operator 3
20. **Respiratory exposures:** Was the operator exposed to high levels of any of the following air contaminants during the past 12 months? (Mark all that apply.)	☐ None<br>☐ Grain/feed/hay dust<br>☐ Animal confinem. dust<br>☐ Field/road dust<br>☐ Manure/silage gases<br>☐ Anhydrous ammonia<br>☐ Fuels/solvents/paints<br>☐ Other, specify: ____	☐ None<br>☐ Grain/feed/hay dust<br>☐ Animal confinem. dust<br>☐ Field/road dust<br>☐ Manure/silage gases<br>☐ Anhydrous ammonia<br>☐ Fuels/solvents/paints<br>☐ Other, specify: ____	☐ None<br>☐ Grain/feed/hay dust<br>☐ Animal confinem. dust<br>☐ Field/road dust<br>☐ Manure/silage gases<br>☐ Anhydrous ammonia<br>☐ Fuels/solvents/paints<br>☐ Other, specify: ____
21. When exposed to high levels of air contaminants at work, what percent (%) of that time did the operator use proper respiratory protection (incl. N95 dust mask, half mask with dust/gas filters)?	Amount of Time (%) ☐☐☐	Amount of Time (%) ☐☐☐	Amount of Time (%) ☐☐☐
22. **Noise exposures:** Was the operator exposed to high levels of noise from any of the following sources during the past 12 months? (Mark all that apply.)	☐ None<br>☐ Tractors<br>☐ Combines<br>☐ Implements<br>☐ Power tools<br>☐ Animals<br>☐ Firearms<br>☐ Other, specify: ____	☐ None<br>☐ Tractors<br>☐ Combines<br>☐ Implements<br>☐ Power tools<br>☐ Animals<br>☐ Firearms<br>☐ Other, specify: ____	☐ None<br>☐ Tractors<br>☐ Combines<br>☐ Implements<br>☐ Power tools<br>☐ Animals<br>☐ Firearms<br>☐ Other, specify: ____

	Principal Operator	Operator 2	Operator 3
23. When exposed to high levels of noise at work, what percent (%) of that time did the operator use proper hearing protection (ear muffs or ear plugs)?	Amount of Time (%)	Amount of Time (%)	Amount of Time (%)
24. **Skin exposures:** Was the operator exposed to any of the following chemicals or animal based allergens while working during the past 12 months? (Mark all that apply.)	☐ None<br>☐ Pesticides/fertilizers<br>☐ Animals/livestock<br>☐ Detergents/disinfectants<br>☐ Fuels/solvents/paints<br>☐ Other, specify:	☐ None<br>☐ Pesticides/fertilizers<br>☐ Animals/livestock<br>☐ Detergents/disinfectants<br>☐ Fuels/solvents/paints<br>☐ Other, specify:	☐ None<br>☐ Pesticides/fertilizers<br>☐ Animals/livestock<br>☐ Detergents/disinfectants<br>☐ Fuels/solvents/paints<br>☐ Other, specify:
25. When exposed to chemicals at work, what percent (%) of that time did the operator use proper personal protection equipment (PPE) (apron, goggles, gloves)?	Amount of Time (%)	Amount of Time (%)	Amount of Time (%)
26. **Musculoskeletal exposures:** Was the operator exposed to any of the following situations at work during the past 12 months? (Mark all that apply.)	☐ None<br>☐ Forceful exertions<br>☐ Repetitive tasks<br>☐ Awkward postures<br>☐ Frequent manual labor<br>☐ Vibration<br>☐ Other, specify:	☐ None<br>☐ Forceful exertions<br>☐ Repetitive tasks<br>☐ Awkward postures<br>☐ Frequent manual labor<br>☐ Vibration<br>☐ Other, specify:	☐ None<br>☐ Forceful exertions<br>☐ Repetitive tasks<br>☐ Awkward postures<br>☐ Frequent manual labor<br>☐ Vibration<br>☐ Other, specify:
27. Was the operator using any of the following preventive techniques to maintain his/her musculoskeletal health during the past 12 months? (Mark all that apply.)	☐ None<br>☐ Regular breaks<br>☐ Stretching<br>☐ Exercising<br>☐ Good lifting techniques<br>☐ Mechanizing tasks<br>☐ Other, specify:	☐ None<br>☐ Regular breaks<br>☐ Stretching<br>☐ Exercising<br>☐ Good lifting techniques<br>☐ Mechanizing tasks<br>☐ Other, specify:	☐ None<br>☐ Regular breaks<br>☐ Stretching<br>☐ Exercising<br>☐ Good lifting techniques<br>☐ Mechanizing tasks<br>☐ Other, specify:

SECTION 7

28. Respondent: ______________________ Date: ___ / ___ / 2020

29. Comments:

THANK YOU FOR COMPLETING THIS SURVEY.

Please review survey results from previous years at: https://www.unmc.edu/publichealth/cscash/

This survey is funded by the Centers for Disease Control and Prevention (CDC) award (U54-OH010162) to the Central States Center for Agricultural Safety and Health which is housed at the University of Nebraska Medical Center.

8299390572
